# Validation of the Arabic version of the breastfeeding behavior questionnaire among Lebanese women

**DOI:** 10.1186/s13006-020-00296-7

**Published:** 2020-06-09

**Authors:** Lama Charafeddine, Saadieh Masri, Lama Shamsedine, Lilian Ghandour, Hani Tamim, Nathalie El Khoury, Zahraa Hachem, Mona Nabulsi

**Affiliations:** 1 Department of Pediatrics and Adolescent Medicine, American University of Beirut, Beirut, Lebanon; 2 Department of Epidemiology and Population Health, Faculty of Health Sciences, American University of Beirut, Beirut, Lebanon; 3 Clinical Research Institute, Biostatistics Unit, American University of Beirut, Beirut, Lebanon; 4 Faculty of Arts and Sciences, American University of Beirut, Beirut, Lebanon

**Keywords:** Breastfeeding behavior, Perceptions, Attitudes, Lebanon

## Abstract

**Background:**

The Breastfeeding Behavior Questionnaire (BBQ) assesses women’s perceptions of their breastfeeding behavior. It was adapted to several languages and used in different settings, but has not been validated in Arabic-speaking populations. None of the previous studies that used the BBQ in other cultures examined its ability to predict the actual breastfeeding behaviors of mothers postpartum. This study validated the BBQ in a cohort of Lebanese pregnant women between December 2013 and January 2016, and examined whether it can predict exclusive breastfeeding at one, three and six months.

**Methods:**

The internal consistency reliability and construct validity of the Arabic BBQ (BBQ-A) were tested on 354 pregnant women. Its predictive ability was assessed by correlating the women’s BBQ-A scores with their breastfeeding outcomes at one, three and six months post-delivery.

**Results:**

The BBQ-A had a good internal consistency reliability (Cronbach’s alpha = 0.78). Exploratory factor analysis revealed that it is unidimensional. Inter-item correlations ranged between − 0.016 and 0.934, with corrected-item total correlations ranging from 0.273 to 0.678. Perceived positive breastfeeding behavior correlated with positive breastfeeding attitudes, good breastfeeding knowledge and stronger breastfeeding intention supporting its external validity. However, in binomial multivariate logistic regression analysis, the BBQ-A did not predict exclusive breastfeeding at one, three or six months.

**Conclusions:**

The BBQ-A is a reliable and valid instrument to assess women’s perceptions of their breastfeeding behavior in an Arab context. Availability of this instrument is important for investigators conducting breastfeeding research in the Arab world. However, the BBQ-A does not predict exclusive breastfeeding at one, three or six months. Further research on the Breastfeeding Behavior Questionnaire is needed to examine its predictive validity in other cultures.

## Background

It is well established that breastfeeding is the most effective public health measure to reduce under-five mortality [1]. Despite the World Health Organization’s (WHO) recommendation for mothers to exclusively breastfeed their infants for six months, and to continue breastfeeding for at least two years [2], the rates of exclusive breastfeeding remain very low worldwide [3], including Arab countries [4–7]. Lebanon has one of the lowest rates of exclusive breastfeeding (15%) in infants below five months of age in the region [8]. Researchers have previously reported that misconceptions about breastfeeding, and social norms that promote formula feeding were major barriers to breastfeeding in Lebanon, negatively influencing mothers’ decisions to initiate or to continue breastfeeding [9, 10]. Lack of motivation to breastfeed is another well-known barrier influenced by multiple psychosocial factors including maternal perceived behavioral control [9, 10]. Based on Ajzen’s theory of planned behavior, perceived behavioral control reflects personal beliefs as to how easy or difficult it is to perform a certain behavior [10]. This theory has been shown to explain health related behaviours, including attitude toward an action and perceived behavioral control, with the latter being a significant predictor of the behavior [11]. Previous literature reported positive associations between predictor variables of the Theory of Planned behavior, such as perceived behavioral control, and breastfeeding behavior [12, 13].

In 1992, Libbus developed the breastfeeding behavior questionnaire (BBQ) to assess women’s perceptions of their breastfeeding behavior [14]. Specifically, the BBQ examines maternal beliefs and attitude towards breastfeeding using 12 different scenarios representing situations that might affect a mother’s breastfeeding choice and practice (e.g., breastfeeding in public and the influence of significant others on her decision to breastfeed) (Additional file 1). The BBQ has been adapted to several languages and used in different settings [14–19]. The questionnaire was translated into Arabic, and used to assess perceived breastfeeding behavior among female undergraduate university students in a previous study from Lebanon [15]. However, the authors did not validate the questionnaire.

The objectives of this study were to: (1) adapt and validate an Arabic version of BBQ (BBQ-A) for the assessment of women’s perceptions of their breastfeeding behavior, (2) assess its ability to predict exclusive breastfeeding at one, three and six months among Lebanese women. The availability of a valid instrument that can assess maternal perceived breastfeeding behavior is essential for breastfeeding research in the Arab countries.

## Methods

### Design

This is an instrument validation study that used secondary data from a larger two-group clinical trial that took place in two tertiary care centers in Lebanon between December 2013 and January 2016 [20]. The trial aimed at investigating whether a complex intervention targeting new mothers’ breastfeeding knowledge, skills and social support within a Social Network and Social Support theory framework would increase exclusive breastfeeding rate and duration among women in Lebanon. In that trial, healthy pregnant women were randomly allocated to either receive standard obstetric care (control group), or receive a breastfeeding promotion and support intervention consisting of peer support, professional lactation support and breastfeeding education, in addition to standard obstetric care (experimental group). Participants in the multi-component intervention group were twice as likely to breastfeed exclusively for six months, compared to standard care (OR 2.02; 95% CI 1.20, 3.39), with the highest odds for exclusive breastfeeding being in participants who complied with all three intervention components (OR 6.63; 95% CI 3.03,14.51) [21].

Trial participants were surveyed about their perceived breastfeeding behavior using the BBQ at baseline and at six months postpartum [20, 21]. Since the BBQ was not validated in the Arabic context, it was deemed necessary to validate the BBQ while conducting the trial. The trial and this instrument validation study were approved by the institutional review boards of both participating centers (PED.MN.08). Written informed consent was obtained from all participants.

### Setting

The trial was conducted in the obstetrics clinics of the two participating centers in the capital of Lebanon between December 2013 and January 2016. Both centers are academic, non-profit, privately-funded tertiary care hospitals. One center serves patients of low and middle income background while the other center serves moderate to high income patients. Antenatal education classes covering labor, delivery and breastfeeding are offered in the latter center only. In both settings, specialized lactation consultants are unavailable, and breastfeeding support is offered mainly by the hospital nurses and doctors.

### Sample

The participants in this validation study were the trial participants. The inclusion criteria were healthy pregnant Lebanese women, in their first or second trimester of pregnancy that intended to breastfeed after delivery, and could read and write in Arabic. Women were excluded if they had a chronic medical condition, did not want to breastfeed, had twin pregnancy, delivered preterm at 37 or less weeks of gestation, had abnormal fetal screen, or did not live in Lebanon for at least six months after delivery. Participants were recruited consecutively as they presented to the outpatient obstetric clinics of the participating centers. Most women who met the trial’s eligibility criteria and were approached for enrolment accepted to participate. The overall number of women approached for enrolment was not recorded but of 446 participants who were consented during pregnancy, 84 (18.8%) withdrew or were withdrawn for various reasons (loss to follow up, premature delivery, fetal death, or neonatal intensive care admission). Of the remaining 362 participants, 22 (6.1%) withdrew prior to completing six months of follow up. There were 345 (95.3%) participants who completed one month, 344 (95.0%) completing three months, and 340 (93.9%) completing six months of follow up [21]. The total sample size for this validation study included 354 participants with complete baseline data. Our participants had demographics that were similar to the general population of Lebanese pregnant women in terms of age, language and ethnicity. However, they differed in education level, income, and area of residence. The majority of our cohort had university degrees, with relatively high income, and lived in the capital city.

### Measurement

The original BBQ has 12 items that describe viewpoints of breastfeeding women that might affect their breastfeeding behavior. Each item assesses the extent of the respondent’s agreement to a specific situation using a 6-point Likert scale that ranges from *Strongly agree* to *Strongly disagree*. The situations concern breastfeeding in front of others, breastfeeding in public places, and the influence of significant others and health care professionals on the decision to breastfeed. The total possible score of the BBQ range from 12 to 72 points. The median of the total BBQ score separates perceived positive behavior from perceived negative behavior, with lower scores representing more positive breastfeeding behaviors than higher scores [14].

After obtaining permission from the primary author (Personal communication, Libbus, 2012), an independent bilingual physician translated the BBQ to classical Arabic. This was because classical Arabic is understood by all Arabic-speaking people. Cultural adaptation and contextualization were done by replacing the names of the persons in the questions by Arabic names, and changing the word “church” to “place of worship”. The questionnaire was back-translated to English by a second independent bilingual translator. Both versions were compared for accuracy by one of the investigators (MN). The Arabic BBQ was found to be an accurate translation of the original BBQ. Hence it was piloted among 20 women visiting in April 2013 the obstetrics clinics at one of the centers, after obtaining their verbal consent. The women had to meet the same inclusion and exclusion criteria of the trial. The participants were asked in an interview to indicate whether the translated questionnaire was clear, easy to understand, of reasonable length, and culturally acceptable (all answers as: Yes/No). The participating women answered “Yes” to all previous attributes. Hence no changes were made to the translated questionnaire (Additional file 2). We did not collect sociodemographic data on the pilot sample, as it was deemed unnecessary given the purpose of the piloting.

### Data collection

At baseline, the participants were administered a questionnaire to collect sociodemographic data (e.g., age, education, employment status, monthly income, religion, gestational age, number of children, number of breastfed children and longest duration of previous breastfeeding in multiparous women). Participants were also administered the following validated Arabic questionnaires as dictated by the larger trial’s protocol: The Infant Feeding Intention-Arabic version (IFI-A) to measure maternal breastfeeding intentions [22]; the Iowa Infant Feeding Attitude Scale-Arabic version (IIFAS-A) to evaluate maternal attitude toward infant feeding methods [23]; the Infant Breastfeeding Knowledge-Arabic version (BFK-A) to assess maternal breastfeeding knowledge [24]; and were surveyed about their perceived breastfeeding behavior using the BBQ.

Information on the infants’ feeding was obtained at one, three and six months by telephone survey, which asked whether the infant was being exclusively breastfed, was on mixed feeding, or on formula feeding. We adopted the WHO’s definition of exclusive breastfeeding, which is feeding the baby human milk only, with no other food or drink including water, but allowing oral rehydrating solutions, vitamins, minerals, or other medicines when needed [25].

### Data analysis

Participants’ baseline characteristics and BBQ scores were summarized as means and standard deviations (*SD*), or medians and interquartile ranges (*IQR*) for continuous variables, as appropriate; and as frequencies and proportions for categorical variables. The BBQ responses were scored in a similar way to the scoring of the original BBQ [14]. We assessed the BBQ’s dimensionality by running an Exploratory Factor Analysis (EFA) on all 12 items using Principal Component Analysis (PCA), with varimax or promax rotation. The Kaiser-Meyer-Olkin (KMO) measure of sample adequacy and Bartlett’s test of sphericity were run to test the suitability of the PCA method. Subsequently, we examined the scree plot and the Eigenvalues to check for the suggested number of factors that the items were loading on. The BBQ’s internal consistency reliability was assessed using Cronbach’s alpha coefficient, as well as its item-total statistics (e.g. item-total correlations and scale reliability coefficient if an item was deleted), to assess whether any items needed to be deleted. After deciding on the final number of factors and items to be retained, a total score was generated for the BBQ responses and recoded as a categorical variable using the median to separate positive from negative score as described by Libbus [17]. BBQ scores equal to or below the median were categorized as suggestive of generally positive breastfeeding behaviors, whereas scores above the median were categorized as suggestive of generally negative breastfeeding behaviors. This method of using the median to split the BBQ score into two categories is a standard statistical technique [26–28], and was used by Libbus and Kolostov in their analyses of the original English BBQ [17].

The associations between the BBQ scores and the different baseline characteristics were assessed using Chi-square for categorical data, and independent Student’s *t*-test for continuous data. The BBQ’s external construct validity was assessed by comparing the participants’ scores on BBQ to their scores on IIFAS-A and BFK-A using Pearson’s correlation coefficient (*r*). These two instruments were chosen because in the original BBQ publication, a positive BBQ score was considered to be indicative of generally positive attitude and accurate knowledge [14]. The predicitve validity of the BBQ was tested in a binomial multivariate logistic regression analysis with *exclusive breastfeeding* (Yes/No) as the dependent variable, and BBQ-A score (forced variable), treatment allocation in the breastfeeding trial (experimental/control), age, education, employment, site, income, mode of delivery, parity, number of breastfed children, and having support at home as predictors. A similar model was built with the same predictors but with *any breastfeeding* (Yes/No) as the dependent variable. All analysis was done using SPSS version 23. Statistical significance was set at a *p* value of < 0.05.

## Results

### Characteristics of the sample

The baseline sociodemographic characteristics of the participants are described in Table 1. The participants’ mean (*SD*) age was 29.3 (4.9) years and their mean (*SD*) gestational age was 17.3 (4.3) weeks. They had a median (*IQR*) number of children of 1.0 (0.0, 1.0), ranging between 0 and 4 children. The number of breastfed children of multiparous mothers also ranged between 0 and 4, with a median (*IQR*) of 1.0 (1.0, 2.0) children. The longest duration of previous breastfeeding in multiparous participants had a skewed distribution with a median (*IQR*) of 0.0 (0.0, 8.3) months.

**Table 1:** Baseline characteristics, breastfeeding behavior scores and breastfeeding outcomes (*N* = 354)

Characteristic	Total *n* (%)
**Allocation**	
Experimental	169 (47.7)
Control	185 (52.3)
**Site**	
Center A	302 (85.3)
Center B	52 (14.7)
**Parity**	
Primiparous	166 (46.9)
Multiparous	188 (53.1)
**Religion**^**a**^	
Muslim	292 (88.2)
Christian	39 (11.8)
**Employment**	
Yes	180 (50.8)
No	174 (49.2)
**Education level**	
≤ Intermediate	36 (10.2)
Secondary or technical	38 (10.7)
University	280 (79.1)
**Monthly income**	
≤ $1000	98 (27.7)
> $1000	256 (72.3)
**Breastfeeding behavior score**	
Positive (12 to 35)	188 (53.1)
Negative (36 to 56)	166 (46.9)
**Breastfeeding behavior**	
EBF at 1 month^a^	175 (49.4%)
EBF at 3 months^a^	140 (39.5)
EBF at 6 months^a^	106 (29.9)

*EBF* Exclusive breastfeeding

^a^Missing values: EBF at 1 month = 16; EBF at 3 months = 17; EBF at 6 months = 21; Religion = 39

The participants’ median (*IQR*) BBQ-A score, which is the cut-off value between positive and negative perceived breastfeeding behavior was 35 (28, 40). The mean (*SD*) scores of the other instruments were 11.2 (2.5) for the BFK-A, 12.4 (3.0) for the IFI-A, and 65.6 (7.2) for the IIFAS-A. The majority (70.3%) of the participants had a neutral attitude towards breastfeeding (IIFAS-A between *mean - SD* and *mean + SD),* and 46.9% had good or very good breastfeeding knowledge (BFK-A score above the mean), while 58.7% of participants had strong intentions to breastfeed (IFI-A score above the mean). The prevalence of any breastfeeding was 90.4% at one month, 71.8% at three months, and 52.8% at six months, which were much higher than the prevalence of exclusive breastfeeding (EBF) at the same time points (Table 1).

### Internal consistency reliability

**Fig. 1:**
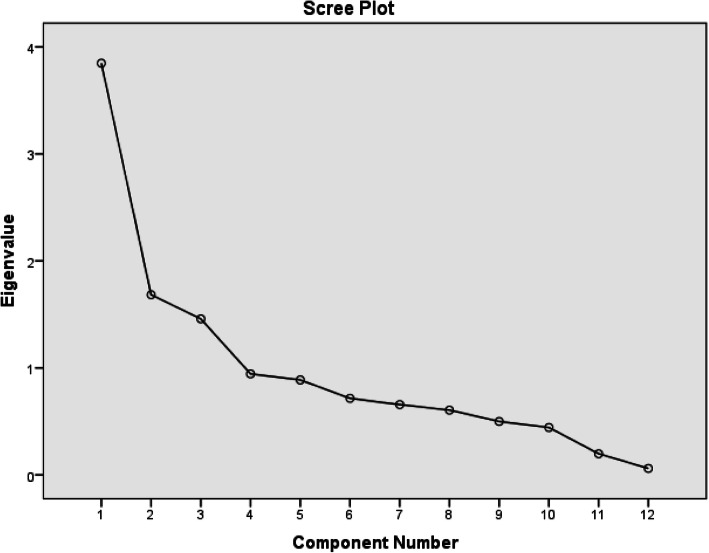
Scree plot of the BBQ-A revealing one point above the curve’s “elbow” (Eigen value of 3.848)

The Cronbach’s alpha internal consistency reliability of the 12-item BBQ was 0.78. Inter-item correlations ranged between − 0.016 and 0.934, with corrected-item total correlations ranging from 0.273 for item 6 to 0.678 for item 7. Cronbach’ alpha if item deleted ranged between 0.78 and 0.74, which implies that none of the BBQ items needed to be re-evaluated or dropped. The KMO measure of sample adequacy for a PCA was 0.774 (*p* < 0.001). Hence, it was considered adequate for PCA [29]. The principal component factor analysis of BBQ (varimax rotation) revealed that it had three components with Eigenvalues above 1, which were 3.848, 1.684 and 1.458. The scree plot (Fig. 1) suggested that it is unidimensional. Table 2 summarizes the psychometric properties of the BBQ-A.

**Table 2:** The psychometric properties of the Arabic Breastfeeding Behavior Questionnaire

BBQ-A item content	Corrected Item-total correlation	Cronbach’s alpha if item deleted
**Item 1:** BF in front of a woman	0.616	0.744
**Item 2:** BF in front of a man and a woman	0.372	0.771
**Item 3:** BF in a restaurant	0.274	0.782
**Item 4:** BF outside a restaurant	0.310	0.776
**Item 5:** BF outside church	0.406	0.766
**Item 6:** BF inside church	0.273	0.783
**Item 7:** Influence of mother on BF behavior	0.678	0.735
**Item 8:** Husband’s influence on BF behavior	0.336	0.773
**Item 9:** Physician’s influence on BF behavior	0.664	0.736
**Item 10:** Artificial milk as good as breast milk	0.418	0.765
**Item 11:** Influence of maternal employment on BF behavior	0.343	0.772
**Item 12:** Artificial milk is better than BF for baby’s weight gain	0.400	0.767

*BF* Breastfeeding

### External validity

Perceived breastfeeding behavior as examined by the BBQ-A did not vary by any of the women’s sociodemographic characteristics. In contrast, the BBQ-A score was significantly and negatively correlated with the IIFAS-A score (*r* = − 0.397; *p* < 0.001), BFK-A score (*r* = − 0.161; *p* = 0.002) and IFI-A score (*r* = − 0.210; *p* < 0.001). This suggests that perceived positive breastfeeding behavior is associated with more positive attitude towards breastfeeding, better breastfeeding knowledge, and stronger infant breastfeeding intention. These correlations support the external validity of the BBQ-A.

The association between BBQ-A score and *exclusive breastfeeding* at one, three and six months was investigated in logistic multivariate analysis to adjust for the allocated intervention in the breastfeeding trial (experimental vs. control), as well other sociodemographic confounders that were associated with EBF on bivariate analysis with a *p* value < 0.1. This analysis revealed that BBQ-A score failed to predict EBF, or any breastfeeding at one, three or six months (Tables 3 and 4).

**Table 3:** The association between participants’ BBQ-A score and exclusive breastfeeding outcomes (*N* = 354)

Breastfeeding Outcome			Crude	Adjusted	
	Positive BB (*n* = 188) *n* (%)	Negative BB (*n* = 166) *n* (%)	*OR (95% CI)*	*OR (95% CI)*	Goodness of fit test *p*
EBF at 1 month	101 (53.7)	74 (44.5)	1.43 (0.93, 2.20)	1.16 (0.73, 1.84)	0.54
EBF at 3 months	80 (42.5)	60 (36.1)	1.31 (0.84, 2.02)	0.97 (0.59, 1.59)	0.95
EBF at 6 months	64 (30.0)	42 (25.3)	1.52 (0.95, 2.43)	1.28 (0.76, 2.16)	0.54

*EBF* Exclusive breastfeeding, *BB* Breastfeeding Behavior, *OR* Odds Ratio, *CI* Confidence Interval

Model adjusted for age (years), treatment allocation (control/experimental), parity (primiparous/multiparous), number of breastfed children (no children/ 1 child/ ≥ 2 children), previous longest breastfeeding duration (months), support at home (yes/no), breastfeeding knowledge score (continuous variable), breastfeeding intention score (continuous variable), and breastfeeding attitude score (continuous variable)

**Table 4:** The association between participants’ BBQ-A score and any breastfeeding outcomes (*N* = 354)

Breastfeeding Outcome			Crude	Adjusted	
	Positive BB (*n* = 188) *n* (%)	Negative BB (*n* = 166) *n* (%)	*OR (95% CI)*	*OR (95% CI)*	Goodness of fit test *p*
ABF at 1 month	170 (90.4)	150 (90.4)	0.88 (0.32, 2.41)	0.81 (0.29, 2.25)	0.65
ABF at 3 months	138 (73.4)	116 (69.9)	1.26 (0.74, 2.14)	0.95 (0.53, 1.72)	0.87
ABF at 6 months	111 (59.0)	76 (45.8)	1.73 (1.09; 2.74)	1.39 (0.82, 2.37)	0.87

*ABF* Any breastfeeding, *BB* Breastfeeding Behavior, *OR* Odds Ratio, *CI* Confidence Interval

Model adjusted for age (years), treatment allocation (control/experimental), parity (primiparous/multiparous), number of breastfed children (no children/ 1 child/ ≥ 2 children), previous longest breastfeeding duration (months), support at home (yes/no), breastfeeding knowledge score (continuous variable), breastfeeding intention score (continuous variable), and breastfeeding attitude score (continuous variable)

## Discussion

There is paucity of validated instruments that measure different aspects of breastfeeding in the Arab context. This study validated the BBQ among a cohort of Lebanese women and provided an Arabic version (BBQ-A) which is a valid useful instrument for investigators researching breastfeeding behavior in Arab countries.

The BBQ-A Cronbach’s alpha coefficient is comparable to the BBQ reliability estimates of 0.64 and 0.89 reported from Kenya [19], and among Hispanic American women [14, 17] using a Spanish BBQ (correlation coefficient of 0.96). Positive BBQ-A responses were associated with positive attitude, better knowledge, and stronger intentions towards breastfeeding. However, the BBQ-A did not predict EBF behavior in our sample. The correlation between perceived breastfeeding behavior as predicted by the BBQ-A, breastfeeding intention and breastfeeding attitude is consistent with the theory of planned behavior in which these variables act as strong predictors of the behavior [11–13]. The fact that the BBQ-A did not predict EBF at one, three or six months is not surprising since external factors such as maternal employment, perceived lack of self-efficacy, perceived milk insufficiency, lack of support from family and friends may negatively influence breastfeeding behavior in the first six months [9, 11–13, 30]. To note, none of the previous studies that used the BBQ in other cultures examined its ability to predict breastfeeding behaviors of mothers postpartum.

Our study has some limitations. Our respondents were recruited from a cohort of women participating in a breastfeeding promotion and support clinical trial [20, 21]. Since one of the trial’s inclusion criteria was “intent to breastfeed”, it may be argued that their views may not be representative of all Lebanese women, some of whom may not have the same enthusiasm for breastfeeding. Although the sociodemographic of our sample is similar to the general population of Lebanese pregnant women in terms of age, language and ethnicity, they differ in the fact that the majority of our cohort were highly educated with university degrees, have relatively high income, and live in an urban area (the capital city). Their perceptions of their breastfeeding behavior therefore may differ from those living in rural areas of the country, those with lesser income, or those who are less educated. Women living in rural areas are economically disadvantaged and therefore may tend to breastfeed more because of cost related reasons. Breastfeeding practices of mothers coming from poorer families in low to middle income countries have been shown to be higher than that of richer families; this has been speculated to be due to the fact that poorer mothers will continue breastfeeding to save on spending [3]. Another limitation relates to piloting of the questionnaire on a small sample of 20 women. It may be argued that this sample may not have captured enough opinions about the questionnaire. However, we believe that this number was sufficient for the piloting phase since all 20 women were recruited from the same participating centers, had the same inclusion and exclusion criteria of the trial, and gave similar evaluations of the questionnaire attributes. Thus, it was deemed unnecessary to revise and re-pilot the questionnaire, or to enlarge the piloted sample size. A third limitation is that Lebanese women who observe the Muslim religion tend to breastfeed more because it is dictated in the Quran that breastfeeding for two years is a mother’s duty. Hence, our sample may represent Arab women living in an urban area but not of the entire Arab women population. There is need therefore to replicate our findings with larger samples, in non-urban settings, as well as in other Arab countries. Despite all these limitations, the BBQ-A proved to be a valid instrument that could be useful for researchers assessing women’s perceptions of their breastfeeding behavior in our context.

## Conclusions

The BBQ-A is a useful tool to assess women’s perceptions of their breastfeeding behavior among Arabic-speaking women but may not predict the actual breastfeeding behavior. Availability of this instrument is important for investigators conducting breastfeeding research in the Arab world.

## Supplementary information

**Additional file 1.** English BBQ.

**Additional file 2.** Arabic BBQ.

## Data Availability

The datasets used and analyzed during the current study are available from the corresponding author on reasonable request.

## Abbreviations

BBQ: Breastfeeding Behaviour Questionnaire
BBQ-A: Breastfeeding Behaviour Questionnaire-Arabic
WHO: World Health Organization
IFI-A: Infant Feeding Intention-Arabic
IIFAS-A: Iowa Infant Feeding Attitude Scale-Arabic
BFK-A: Breastfeeding Knowledge-Arabic
EFA: Exploratory Factor Analysis
PCA: Principal Component Analysis
KMO: Kaiser-Meyer-Olkin
SD: Standard deviation
IQR: Interquartile range
EBF: Exclusive breastfeeding
