# Supplementary material for: Validation of the Arabic version of the breastfeeding behavior questionnaire among Lebanese women
Source: Int Breastfeed J. 2020 Jun 9;15:54. doi: 10.1186/s13006-020-00296-7 (PMC7282113; doi:10.1186/s13006-020-00296-7)
Supplement: Supplementary file 1 — Additional file 1. English BBQ. [file 13006_2020_296_MOESM1_ESM.pdf]

<u>Breastfeeding Behavior Questionnaire (BBQ)</u>

Adapted with permission from Libbus MK (Personal communication, 2012).

DIRECTIONS: For each of the following scenarios, participants are asked if they agree or disagree with the woman's choice using a 6-point Lickert-scale.

VSD= very strongly disagree
SD = strongly disagree
D= disagree
A=agree
SA= strongly agree
VSA= very strongly agree

1. Jane Johnson, a new mother, is breastfeeding her baby in the living room. Her girlfriend from next door comes to see the new baby. Jane covers her breast and the baby's head with a receiving blanket and the baby continues to nurse while the two women talk. Do you agree that it was all right for Jane to continue breastfeeding?

2. Estelle Green is breastfeeding her baby in the living room. The man and woman from next door come to see the new baby. Estelle covers her breast and the baby's head with a receiving blanket and the baby continues to breastfeed while the neighbors talk. Do you think Estelle should have stopped breastfeeding?

3. Martha Smith is at McDonald's eating lunch with her girlfriends. When her baby wakes up and seems hungry, she decides to breastfeed him under her blouse. Do you think Martha should have taken the baby out of the public place to breastfeed?

4. Kathy Brown is eating lunch at Dairy Queen with her girl friends. When her baby wakes up and seems hungry, she decides to breastfeed him under her blouse. Her friends are embarrassed by this, so she takes him out to the car to breastfeed him instead. Do you agree with Kathy's decision to take the baby out to the car to breastfeed him?

5. Anne Evans and her husband take their baby to church. When it is time for the baby to breastfeed, Anne takes her into the ladies' bathroom. Do you think it was necessary for Anne to take the baby out of church to breastfeed?

6. Marie Schultz and her husband take their baby to church. When it is time for the baby to eat, Marie breastfeeds the baby under her blouse. She also covers the baby's head with a receiving blanket in case the blouse slips. Do you think that Marie should have taken the baby out of church to breastfeed?

7. June Moon is expecting her first baby and wants to breastfeed. June's mother tells her that no one in their family has been able to successfully breastfeed since all the women have small breasts and can't make enough milk. June decides to breastfeed anyway. Do you agree with June's decision?

8. Laura Baxter is expecting her first baby and wants to breastfeed. Laura's husband wants her to bottle-feed the baby because he says that breastfeeding is "embarrassing." Laura decides to bottle-feed instead of breastfeeding. Do you agree with Laura's choice to not breastfeed because of her husband's opinion?

9. Linda Martin is pregnant and her doctor tells her that she should plan to breastfeed her new baby. Linda had planned to bottle-feed but changes her mind. Do you agree with Linda's decision to follow her doctor's advice?

10. Jane Blaine, who is expecting her first baby, was advised to breastfeed her new baby because "human milk is better for human babies." Jane decides to bottle-feed instead because she has heard that formula is every bit as good as breastmil Do you agree with Jane's decision to *not* breastfeed her baby?

11. Peggy Kelly is expecting her first baby very soon. She was advised to breastfeed but decides to bottle-feed because she wants to go back to work when the baby is 3 months old and has heard that a breastfed baby won't take a bottle. Do you agr with Peggy's decision *not* to try to breastfeed her baby?

12. Jeanette James is expecting her second baby. Even though she has been told that breastfeeding is better for babies, she decides to bottle-feed. She tried to breastfeed her first baby and had to stop because the baby lost weight during the first week. Do you agree with her decision to *not* try breastfeeding this baby?
