# Supplementary material for: Validation of the Arabic version of the breastfeeding behavior questionnaire among Lebanese women
Source: Int Breastfeed J. 2020 Jun 9;15:54. doi: 10.1186/s13006-020-00296-7 (PMC7282113; doi:10.1186/s13006-020-00296-7)
Supplement: Supplementary file 2 — Additional file 2. Arabic BBQ. [file 13006_2020_296_MOESM2_ESM.pdf]

إستبيان التصرف فى الرضاعة الطبيعية

Breastfeeding Behavior Questionnaire (BBQ)

التوجيهات: لكل من هذه السيناريوهات تسأل المشتركات ما إذا كن يوافقن أو لا يوافقن مع خيار المرأة عبر إستخدام مقياس لايكرت Lickert Scale ذا النقاط الست:

1= موافقة بشدة أكثر 2= موافقة بشدة 3= موافقة 4= غير موافقة 5= غير موافقة بشدة
6= غير موافقة بشدة أكثر

1. لينا أم جديدة ترضع طفلها من ثديها في غرفة المعيشة. صديقتها من الباب المجاور تأتي لرؤية الطفل الجديد. لينا غطت ثديها ورأس الطفل ببطانية، وإستمر الطفل يرضع في حين كانت المرأتان تتحدثان. هل توافقين أنه لا إشكال في إستمرار لينا بالرضاعة الطبيعية؟

6 5 4 3 2 1

2. مايا ترضع طفلها من ثديها في غرفة المعيشة. جاء الرجل والمرأة من الباب المجاور لرؤية الطفل الجديد فغطت مايا ثديها ورأس الطفل ببطانية وبقي الطفل يرضع في حين كان الجيران يتحدثون. هل تعتقدين أنه كان على مي أن توقف الرضاعة؟

6 5 4 3 2 1

3. هدى في الماكدونالد تتناول طعام الغداء مع صديقاتها. عندما إستيقظ طفلها وبدا جائعا" قررت إرضاعه من تحت قميصها. هل تعتقدين أنه كان ينبغي على هدى أن تأخذ طفلها بعيدا" عن المكان العام لإرضاعه؟

6 5 4 3 2 1

4. غادة تتناول طعام الغداء في المطعم مع صديقاتها. عندما إستيقظ طفلها وبدا جائعا" قررت أرضاعه من تحت قميصها. أحست صديقاتها بالإحراج فأخذت طفلها إلى السيارة لإرضاعه. هل توافقين على قرار هدى أن تأخذ طفلها إلى السيارة لإرضاعه؟

6 5 4 3 2 1

5. إصطحبت حنان وزوجها طفلتهما إلى دار العبادة. عندما حان وقت إرضاعها أخذت حنان طفلتها إلى حمام السيدات. هل تعتقدين أنه كان من الضروري لحنان أن تأخذ طفلتها إلى خارج دار العبادة لإرضاعها؟

6 5 4 3 2 1

6. أصطحبت نور وزوجها طفلهما إلى دار العبادة. عندما حان وقت إرضاعه أرضعته نور من تحت القميص، وغطت أيضا" رأس طفلها ببطانية في حال إنزلق قميصها. هل تعتقدين أنه كان ينبغي على نور أن تأخذ طفلها إلى خارج دار العبادة لإرضاعه؟

1 2 3 4 5 6

7. جون مون تنتظر مولودها الأول وتريد إرضاعه رضاعة طبيعية. والدة جون تخبرها أن لا واحدة من عائلتهم نجحت بالرضاعة الطبيعية لأن كل نساء العائلة لديهن أثداء صغيرة ولا تنتجن كميات حليب كافية. تقرر جون ممارسة الرضاعة الطبيعية على أي حال. هل توافقين جون الرأي؟

1 2 3 4 5 6

8. لميس تنتظر مولودها الأول وتريد إرضاعه طبيعيا". زوج لميس يريدها أن تعطي الطفل الحليب المجفف للأطفال لأنه يقول أن الرضاعة الطبيعية محرجة. تقرر لميس أن تعطي طفلها الحليب المجفف للأطفال بدل الرضاعة الطبيعية. هل توافقين لميس على خيارها بعدم ممارسة الرضاعة الطبيعية بسبب رأي زوجها؟

1 2 3 4 5 6

9. منى حامل وأخبرها طبيبها أنها يجب أن تخطط لإرضاع طفلها رضاعة طبيعية. كانت منى قد قررت أن تغذي طفلها بحليب الأطفال المجفف لكنها تغير رأيها. هل توافقين منى غلى قرارها بإتباع نصيحة الطبيب؟

1 2 3 4 5 6

10. وداد التي تنتظر مولودها الأول تلقت نصيحة بأن ترضع طفلها الجديد من ثديها لأن "حليب الإنسان هو الأفضل لطفل الأنسان". قررت وداد أن تغذي طفلها بحليب الأطفال المجفف لأنها سمعت أنه بجودة حليب الأم. هل توافقين وداد على قرارها بعدم ممارسة الرضاعة الطبيعية؟

1 2 3 4 5 6

11. سوزي تنتظر مولودها الأول قريبا" جدا". تلقت نصيحه بأن ترضعه من ثديها لكنها قررت أن تغذي طفلها بحليب الأطفال المجفف لأنها تريد العودة إلى العمل عندما يبلغ طفلها الثلاثة أشهر من العمر. وكانت سمعت أن الطفل الذي يرضع من الثدي لن يتناول قنينة الحليب المجفف. هل توافقين على قرار سوزي بعدم محاولة إرضاع طفلها من الثدي؟

1 2 3 4 5 6

12. ديما تنتظر مولودها الثاني. على الرغم من أنها سمعت أن الرضاعة الطبيعية أفضل للأطفال إلا أنها قررت أن تغذي طفلها بحليب الأطفال المجفف. حاولت أن ترضع طفلها الأول من الثدي لكنها أضطرت للتوقف لأنه خسر وزنا" خلال الأسبوع الأول. هل توافقين ديما على رأيها بعدم محاولة إرضاع طفلها رضاعة طبيعية؟

1 2 3 4 5 6
